# Two systems drive attention to rewards

**DOI:** 10.3389/fpsyg.2014.00046

**Published:** 2014-02-05

**Authors:** Christopher K. Kovach, Matthew J. Sutterer, Sara N. Rushia, Adrianna Teriakidis, Rick L. Jenison

**Affiliations:** ^1^Department of Neurosurgery, Carver College of Medicine, University of Iowa Hospitals and ClinicsIowa City, IA, USA; ^2^Department of Neurology, Carver College of Medicine, University of Iowa Hospitals and ClinicsIowa City, IA, USA; ^3^Department of Psychology, University of Wisconsin–MadisonMadison, WI, USA

**Keywords:** attention, eye movements, decision making, reward processing, framing effect, decision bias, approach/avoidance

## Abstract

How options are framed can dramatically influence choice preference. While salience of information plays a central role in this effect, precisely how it is mediated by attentional processes remains unknown. Current models assume a simple relationship between attention and choice, according to which preference should be uniformly biased towards the attended item over the whole time-course of a decision between similarly valued items. To test this prediction we considered how framing alters the orienting of gaze during a simple choice between two options, using eye movements as a sensitive online measure of attention. In one condition participants selected the less preferred item to discard and in the other, the more preferred item to keep. We found that gaze gravitates towards the item ultimately selected, but did not observe the effect to be uniform over time. Instead, we found evidence for distinct early and late processes that guide attention according to preference in the first case and task demands in the second. We conclude that multiple time-dependent processes govern attention during choice, and that these may contribute to framing effects in different ways.

## INTRODUCTION

How the human brain selects beneficial actions under biologically determined limits of processing is a central question in the study of decision behavior. Classical economics considers choice from the standpoint of a rational actor, whose behavior optimally maximizes gain and minimizes loss, abstracting away many of the ecological factors that influence choice in the real world. The historical roots of psychiatry, on the other hand, lie in disentangling the complex array of motives that drive behavior, which seems often as not to defy any rational account. More recently, scientific focus has centered on the importance of constraints on rational decision-making grounded in the requirements of biological computation in the real world (Simon, 1955), giving rise to a large and highly influential body of literature at the intersection of economics and psychology (Simon, 1956; Kahneman, 2011). Two core insights to come from this work are that logically extraneous information frequently influences our choices, giving rise to so-called framing effects (Tversky and Kahneman, 1981), and that behavior often appears to be guided not by a unitary rational actor but by multiple distinct systems, which apply differing strategies of learning and response generation in different contexts (Daw et al., 2005).

Framing effects depend in large part on the salience of information that bears on a decision, which in turn depends on the manner in which a choice is presented (Shafir, 1993). Attentional processes therefore must play a central role in framing, but precisely which processes and what roles remain open questions. Studies that examine framing effects introduce an external, if implicit, manipulation of attention, yet the origin, time-course and other details of this process in the context of decision-making have not been clearly delineated.

Studies that consider the relationship between choice and attention by measuring patterns of eye movements leading up to a decision have found that gaze gravitates increasingly towards the item ultimately chosen. Two models explain this effect in different ways. The “gaze cascade model” (Shimojo et al., 2003) supposes that preference and attention exert a bidirectional influence on each other with attention gravitating towards the preferred option, as the attended option also becomes more preferred through the “mere exposure effect”(Zajonc, 1968), resulting in a self-reinforcing cycle. A more recently proposed model explains the relationship through the enhancement of inputs from the attended option in biasing the direction of a drift-diffusion process, (Krajbich et al., 2010). In contrast to the gaze cascade model, this model assumes no reverse influence of preference on attention. Both models predict that external manipulations of attention should alter choice outcome, reminiscent of framing effects, and some evidence bears out these predictions (Armel et al., 2008). To date, however, no model offers a detailed account of how framing influences attention over the course of a decision.

We hypothesized that framing would influence the evolution of gaze culminating in a simple choice. To test this hypothesis, framing was manipulated during a forced choice between two visually presented foods. Subjects selected the preferred item to keep (“keep frame”) in one instance and the less preferred item to discard in the other (“discard frame”). Eye movements, recorded continuously over the course of the trial, provided a time-specific online measure of attention. Our framing manipulation allowed us to distinguish separate contributions of preference and task demands in guiding attention. The latter reflected the so-called response compatibility (Fitts and Seeger, 1953) of the stimulus : subjects directed their gaze more to the chosen item during the keep frame and the discarded item during the discard frame. This finding appears to preclude a simple relationship between attention and preference, as the less preferred item was more attended in the discard condition. We also observed two moment-by-moment patterns that challenge the assumption of a single process, which biases choice and attention uniformly in one direction or another, as suggested by the models previously mentioned (Shimojo et al., 2003; Krajbich et al., 2010). Our findings suggest instead that eye movements are influenced both by preference and by framing, each with a distinct and non-overlapping time course. In both conditions, gaze was rapidly and transiently directed to the preferred stimulus within 500 ms, followed by a monotonically growing bias towards the response-compatible item. We interpret these two phases as evidence for the influence of multiple processes on attention over the course of a decision, with preference-driven responses dependent on a more rapid and transient process. We consider the possible implications of our findings for the origin of framing effects in choice behavior, which may be explained by multi-process accounts.

## MATERIALS AND METHODS

### STIMULI

All stimuli are composed of images selected from a set of 77 foods. The stimulus set, identical to those used by Krajbich et al. (2010), contains items expected to be appetitive for the subject, such as common snack foods. In the rating block, all 77 stimuli were presented. In the subsequent choice blocks pairs of stimuli were selected from those given a positive rating on the rating task.

#### Rating task

Subjects were sequentially presented with all 77 images of food items on an LCD monitor. Using a slider with 20 tick marks labeled -10 to 10, subjects were instructed to rate each item on how much the he or she liked the item, where -10 indicated strong dislike and +10 strong liking.

#### Stimuli for the choice tasks

Following the rating block, the difference of ratings was computed among all pairs of positively rated stimuli. Each pair was assigned a weighting score computed as the inverse of the overall frequency of stimulus pairs with the given difference. Pairs of stimuli were selected iteratively in proportion to weighting in order to achieve an approximately uniform distribution of differences across the range of -5 to +5. Weight on selected pairs was decreased at each selection in order to decrease the likelihood of repeating pairs. This procedure was designed after that of Krajbich et al. (Krajbich et al., 2010).

#### Experiment 1

Subjects were presented 275 randomly selected pairs of positively rated food items across two blocks and asked to choose one item from the pair. In both blocks, participants viewed a fixation cross for a variable 500–1000 ms period after which a pair of food items was presented. After viewing the two options for 1000 ms, the participant was prompted with the question “Which do you prefer?” and selected with a button press one snack to keep. Following the response, the selected item was highlighted. The procedure follows the method and stimulus set used by Krajbich et al. (2010) with the addition of a delayed response window.

#### Experiment 2

Results of Experiment 1 led us to develop a second version of the task, which introduced manipulations of framing and response-cue delay. As in Experiment 1, subjects were presented 275 randomly selected pairs of positively rated food items and asked to choose one item from the pair. A second group of subjects participated in this experiment.

***Variable response-cue delay.*** In order to rule out the possibility that the gaze response observed in Experiment 1 reflected the fixed timing of the response cue, which appeared at 1000 ms, a variable delay of 750–1750 ms was added between the appearance of the choice stimulus and response cue.

**FIGURE 1:**
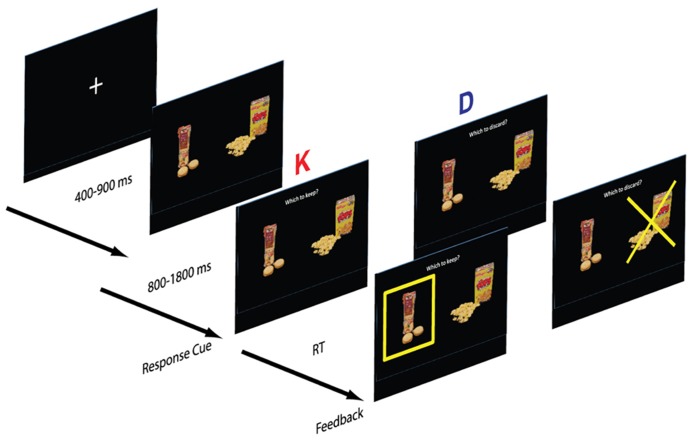
**Structure of the task.** Subjects were cued to respond after a 1 s delay from the appearance of the stimuli. In the “keep” version (K), subjects selected the preferred item and in the “discard” version (D), subjects discarded the less preferred item. The small food images are modified from the stimulus set developed by Ian Krajbich and Antonio Rangel and shared with us.

***Framing manipulation.*** Each subject completed two blocks, a keep block (K) and discard block (D). In the keep block, participants viewed a fixation cross for a variable 500–1000 ms period, after which a pair of food items was presented. After viewing the two options for a variable period, the participant was prompted with the question “Which one to keep?” and selected one snack to keep with a button press. The chosen item was highlighted with a yellow box. In the discard version participants were instead prompted with the question “Which one to discard?” and selected one food item to discard (**Figure 1**). Following the response, the discarded item was highlighted with a superimposed yellow “X”. Subjects were informed that at the end of the task they would receive the chosen food from a randomly selected trial.

### SUBJECTS

For Experiment 1, 20 individuals were recruited from the University of Iowa Hospitals and Clinics community (seven male, 13 female). Ages ranged from 20 to 64, with a mean age of 38. For Experiment 2, a second sample of 23 individuals was recruited (8 male, 15 female). Ages ranged from 19 to 63, with a mean age of 34. All subjects provided voluntary informed consent in accordance with the requirements of the University of Iowa Biomedical Internal Review Board and regulations of the US Department of Health and Human Services before participating in any experiment.

### ACQUISITIONS AND PREPROCESSING OF GAZE DATA

Eye movements were monitored with a remotely mounted infrared video eye tracker (Eyelink 1000, SR Research, Kanata, ON, Canada). Eye position data are sampled at 1 kHz, and fixation onsets were determined using a velocity threshold criterion as implemented in Eyelink software. Further data analysis was carried out using custom developed scripts in Matlab (Mathworks, Nattick, MA, USA).

To analyze gaze data in the decision task, we treated gaze position as a binary variable, indicating fixation to the left or right item. As the distribution of fixations typically showed distinct modes associated with the left and right stimulus and fixation cross, a mixture of Gaussians (MOG) with three component distributions was fitted to the horizontal screen coordinate, and fixations were classified as left, right or middle if the responsibility weight for the corresponding distribution exceeded 0.7. This data-driven approach to classification was chosen over a simpler region-of-interest-based method in order to improve robustness to calibration error in the gaze position. Middle fixations and fixations for which the responsibility weight did not exceed 0.7 for any of the three distributions were discarded from subsequent analysis. Fitted distributions were visually inspected against the histogram of fixation positions in order to ensure that MOG optimization converged appropriately. In the case of failed convergence, starting parameters of the component Gaussians were adjusted manually until fitting converged successfully. After classification, fixation data were converted to a time series in which rightward fixations were coded as +1, leftward as 0 and discarded fixations or periods of signal dropout as NaN.

### GENERALIZED LINEAR MODEL (GLM) ANALYSIS

We applied a regression analysis to distinguish multiple distinct factors influencing the gaze response, including effects of preference and framing condition. A logistic regression with gaze direction as the dependent measure (right = 1 vs. left = 0) modeled the main effect of preference and interactions of preference with framing condition. The GLM included additional interactions between preference and response time and cue onset delay in order to determine whether these variables contributed to the observed effects. The model was fit separately at each time point in the trial relative to stimulus onset. This model treats the log-odds, η_i_[*t*], of a rightward fixation at time *t *on trial *i *as a linear function of the inputs:

(1) $$\eta_{i}\left[t\right]=\beta_{0}\left[t\right]+\Delta r_{i}\times \left(\beta_{1}\left[t\right]+\beta_{2}\left[t\right]B_{i}+\beta_{3}\left[t\right]RT_{i}+\beta_{4}\left[t\right]CD_{i}+\beta_{5}\left[t\right]B_{i}RT_{i}+\beta_{6}\left[t\right]B_{i}CD_{i}\right)$$

Where

(2) $$\Delta r_{i}=r_{i}^{R}-r_{i}^{L}$$

is the difference between ratings for right and left stimuli, *B*_i_ indicates the block type

$$B_{i}=\left\{ \begin{array}{cc} +1 & for\,\;keep\,\;\,\;block\\ -1 & for\,\;discard\,\;block \end{array}\right.$$

*RT*_i_ is the *z*-scored response time and *CD*_i_ is the response cue delay centered at 1.3 s.

The first term in the model is the intercept, capturing a tendency to look either left or right irrespective the stimulus. All other modeled terms represent the main effect of rating difference and its interactions.

#### Model fitting

The model was fit separately for each participant and time point using maximum likelihood estimation as implemented in the GLMFIT function in the Matlab Statistics Toolbox.

#### Population-level analysis

Population-level effects were evaluated with *t*-tests applied to individually fitted parameters at each time sample.

#### Data exclusion

**FIGURE 2:**
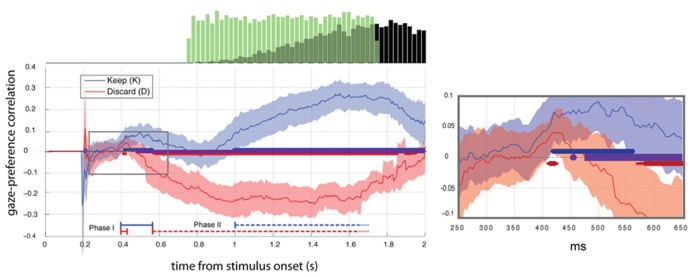
**Gaze preference effect aligned to stimulus onset (*t* = 0).** Mean ±2 SEM gaze-preference correlation curve for each condition (*N* = 23). Thick blue and red lines at the *x*-axis indicate significant deviations from zero (FDR corrected *Q* < 0.05). Periods of significant difference between blocks are indicated with the purple line. Right: Area of the expanded plot (right) is indicated with the gray box. Histograms (top) show the distributions of cue onsets (green bars) and responses (black bars).

Data at any time point for which fixation position was not sorted into one of the two distributions of interest were treated as missing. Because in some cases a paucity of data at a given time sample resulted in excessively large estimation error, and hence inflated magnitude in the estimates of the model parameters, a given individual’s parameter estimate was excluded in the population-level analysis if fewer than 50 data points (37% of trials) were used in fitting the model. Because typically gaze fell on a central fixation cross at the outset of the trial, the model could not be fit for any individual before the time of the first fixation, roughly 200 ms. This limitation is reflected in the absence of the curves before 200 ms in **Figures 2 and 4**.

#### Time contrasts

Additional contrasts were computed over pairwise differences between parameter estimates at each time point. The aim of this analysis was to give a more detailed representation of the time-course of the gaze response, showing when differences with respect to time were significance.

## RESULTS

### EXPERIMENT 1

In Experiment 1, we measured the influence of food preference on gaze direction with the correlation between fixation side and the difference of preference ratings between choice options. This correlation was computed at each time sample with respect to onset of the trial, giving the time-varying gaze-preference curve. In the average across subjects (*N* = 20), we observed distinct early and late periods of significant positive association (unpaired two-tailed *t*-test, FDR corrected *Q* < 0.05) between pre-trial preference rating and gaze within. The early phase lasted from 400 to 600 ms and the late phase extending beyond 900 ms. As the results for Experiment 1 closely matched those for the keep condition of Experiment 2, they are not separately displayed.

### EXPERIMENT 2

To examine the association between gaze and choice we computed Pearson’s correlation between gaze position and preference rating for each time-point in the trial, excluding discarded time points (NaNs). **Figure 2** shows average correlation ±2 SEM across subjects in Experiment 2. Time points differing significantly from 0 (*t*-test, FDR corrected *Q* < 0.05) are indicated with thick lines along the abscissa.

#### Gaze-preference effect in the keep condition

In Experiment 2 we verified the biphasic structure of the response seen in Experiment 1 and observed its dependence on framing and response cue timing. During the keep condition two distinct phases of significant positive correlation (*t*-test, FDR *Q* < 0.05) between preference and gaze response appeared in the average (*N* = 23) with an early period of significant positive correlation between 420 and 560 ms and late period from 1000 ms, replicating the result of Experiment 1 (**Figure 2**).

#### Gaze-preference effect in the discard condition

During the discard block, we saw a brief significant gaze bias toward the non-discarded (i.e. preferred) item at 400–425 ms (Phase I). This was followed by a phase of increasing bias toward the discarded item beginning at 575 ms (Phase II). In both conditions we therefore observed an early phase of gaze bias towards the more highly rated item and a second phase towards the more response-compatible item.

#### Comparison between keep and discard conditions

No statistically significant difference between conditions was observed within the first 450 ms, later than the onset of phase I in both cases, thus we find no evidence for a difference of the onset times for the phase I response across the conditions. In contrast, the duration of the phase I response was greatly diminished in the discard condition, with responses clearly diverging between 450 and 500 ms (paired *t*-test, FDR *Q* < 0.05) and remaining different throughout the rest of the trial. This difference reflected the delayed onset of the phase II response in the keep condition, which began 425 ms later than in the discard condition.

#### Time contrasts

**FIGURE 3:**
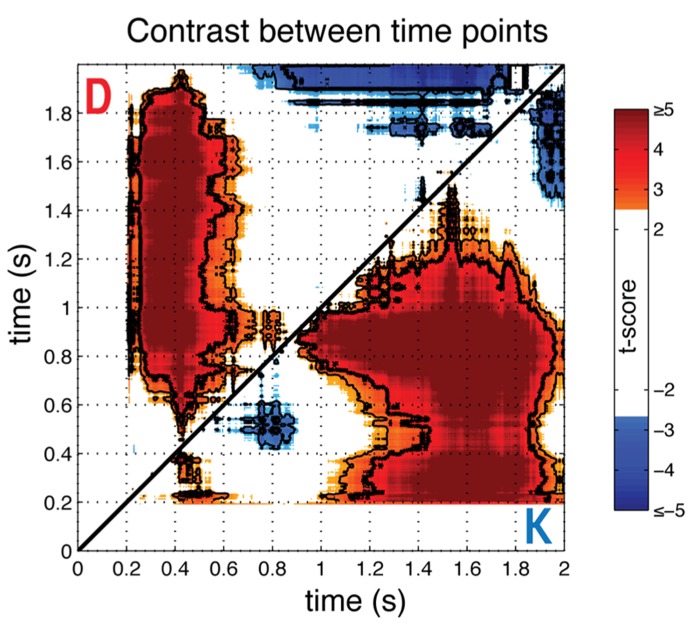
**Comparison across time points.** To show the significance of non-monotonicity in block K and the differences in the bias towards the response-compatible item across blocks, the pairwise difference between time points for individual gaze-preference curves was computed and averaged across individuals (*N* = 23), shown as a *t*-score. Results for the keep condition are below the diagonal (K), and discard condition above (D). Plots are thresholded at FDR corrected significance of *Q* < 0.1 and significance levels of *Q* < 0.05 and *Q* < 0.01 are indicated, respectively, with thin and thick contour lines. Each point represents a t-score for the paired difference between the gaze-preference effect at the time indicated at the abscissa minus the effect at the time indicated at the ordinate, with respect to stimulus onset.

To provide more direct statistical evidence for the time-dependence of the gaze-preference effect, we calculated the average (*N* = 23) differences in individual gaze preference curves between each pair of time samples with respect to stimulus onset (**Figure 3**). In particular, we wished to observe whether pair-wise contrasts between time-points supported the differences in direction and monotonicity between the K and D blocks suggested by **Figure 2**. This analysis verified the non-monotonicity of the gaze-preference curve in the K version of the task, with the magnitude of the average correlation in phases I and II differing significantly from intervening period of non-significant correlation around 800 ms (paired *t*-test, FDR *Q* < 0.05).

### GLM RESULTS

To more clearly distinguish the separate influences of preference and task as well as other potentially confounding factors we followed up the preceding analysis with a logistic regression on the data from Experiment 2. Gaze direction served as the dependent measure with task and preference as the main covariates of interest. Results given here are the averages of individual subjects’ parameter estimates. This analysis revealed the following effects.

#### Effect of preference rating and framing conditions

**FIGURE 4:**
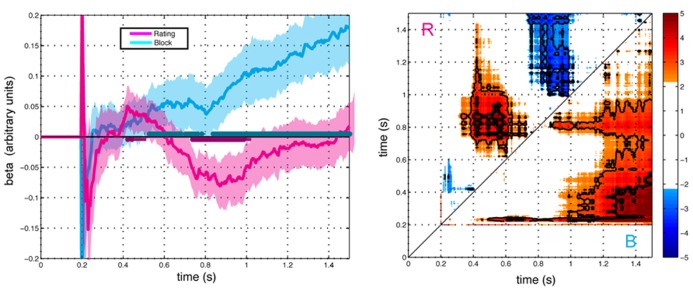
**Effects of preference rating (magenta) and block (cyan) terms on gaze direction as modeled in the GLM with respect to stimulus onset (*t* = 0).** Left Panel: Average beta values ±2 SEM over time for each term (*N* = 23). Right Panel: Paired contrasts between time points for the preference rating term (R, above the diagonal) and the block term (B, below the diagonal). Color scale shows t-scores for the paired contrast between time points thresholded at FDR corrected significance level *Q* < 0.1. Regions for *Q* < 0.05 and *Q* < 0.01 are indicted with thin and thick contour lines, respectively. Points represent the t-score for the paired difference between rating parameter at the time indicated at the abscissa minus the time indicated at the ordinate.

**Figure 4** shows parameter estimates averaged across subjects for the influence of framing and preference on gaze direction. The effect of preference exhibited a biphasic pattern with an initial positive loading followed by a negative loading (FDR *Q* < 0.05). In contrast, the loading on framing condition increased monotonically. Consistent with the correlation analysis, the combined effect of framing and preference yielded a biphasic bias of gaze towards the preferred item in the K condition and an initial bias toward the preferred item followed by a monotonically increasing bias towards the less preferred (more response-compatible) item in the D condition. The regression analysis separates the effect into distinct contributions of preference and framing, revealing former to be characterized by an early positive influence between 400 and 500 ms, followed by a reversal of the effect between 700 ms and 1000 ms. The contribution of framing was characterized by monotonically increasing influence with a relatively later onset at 500 ms.

#### Additional terms

**FIGURE 5:**
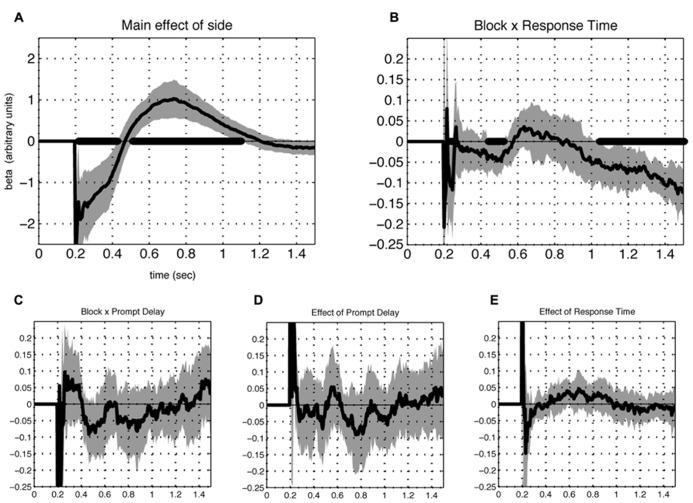
**Contribution of additional terms of the model.** A significant (FDR corrected *Q* < 0.05) effect of the intercept term reflects a prominent tendency for subjects to scan left to right **(A)**. Another term that contributed significantly was the interaction between preference (rating difference), block type and response time **(B)**, for which a negative value appeared separate in early and late phases, meaning that reaction time was reduced when the fixations were directed at the preferred item in the K block and the less preferred item in the D block during those periods. Additional terms representing the interactions between preference and **(C)** the block-by-cue-delay interaction **(D)** prompt cue delay and **(E)** reaction time, were non-significant. In all cases, lines indicate beta values averaged over individuals (*N* = 23). Shaded areas represent 2 SEM and time points which differed significantly from 0 at FDR corrected *Q* < 0.05 are indicated with thick black line at the abscissa.

Of the additional terms included in the model, significant effects appeared for two. First, the main effect of laterality (**Figure 5A**) revealed a prominent tendency to scan items left-to-right. Second, a significant three-way interaction between rating, framing condition and reaction time emerged during both phase I and II, but not the intervening period between them (**Figure 5B**). This effect implies that gaze directed towards the response-compatible item correlated with speeded reaction during both phases.

Additional terms of the model did not show significant effects. These included the interactions of rating and reaction time, rating and prompt delay, and the three-way interaction of rating, prompt delay, and framing condition (**Figures 5C–E**). The latter observation suggests that the appearance of the response cue had little influence on the gaze response and is therefore unlikely to contribute to the observed phasic pattern.

## DISCUSSION

Our results show two qualitatively different phases in the time course of attentional bias during choice, supported both by the bimodal profile of the response in the keep condition and the differing effects of the keep and discard frame on the two phases (**Figure 2**). The regression analysis shown in **Figure 4** distinguishes a transitory bias towards the preferred item associated with phase 1 from a graded monotonic bias towards the response-compatible item in phase 2. These observations suggest the influence of two processes, the more rapid of which, process 1, biases attention towards the preferred option, while process 2 directs attention according to response compatibility. Thus we provide new evidence of separable processes corresponding to early and late orienting during decision-making as well as evidence for the contribution of those processes to attentional consequences of framing.

These results support a dual-system account of orienting and action selection (Kahneman, 2011). The early phase of gaze-bias in the “keep” condition occurs rapidly, reflecting the influence of process 1 on attention towards the motivationally more salient item. In contrast, the second phase of gaze-bias in the “keep” condition occurs later in the trial. The later and more framing-specific contribution of process 2 regulates attention according to the particular action required in given a context. The phases emerge with non-overlapping time courses, suggesting they may be mutually inhibitory. That is, to effectively engage either process requires suppressing any competing outputs of the other.

Dual system models have become commonplace in multiple areas of psychology, featured in accounts of both attention and decision-making. Dual processes of visual orienting and perception (Bar, 2003) to biologically salient stimuli such as faces (de Gelder and Rouw, 2001; Vuilleumier, 2005) have been widely discussed. Rapid orienting serves an obvious ecological function, allowing an organism to respond quickly to threatening conditions (Sokolov, 1963; Eastwood and Smilek, 2005). In contrast, despite the interest in the role of dual systems in decision biases (Kahneman et al., 1990), comparatively little is understood about the ecological origins of such biases (McDermott et al., 2008). In the present case it is reasonably apparent how one might benefit from a system tuned to rapidly and efficiently identify appetitive stimuli in the environment, and while our study was not designed to uncover framing effects in subjects’ pattern of choices, the work of Shafir on the effect of keep and discard frames has shown that salience of information, and by implication mechanisms of attention, play a role in classical framing effects revealed by choice (Shafir, 1993). By linking the attentional effects of framing to multiple systems, we lay a foundation for a more detailed ecological account of framing effects.

In a study which applied a similar manipulation of framing, Shafir presented subjects with a choice between an “impoverished” and an “enriched” option, with the latter having more extreme positive and negative attributes than the former. He found that choice gravitated towards the enriched option in the keep frame and the impoverished option in the discard frame. This he related to compatibility effects, the well established observation that response difficulty is affected by how naturally responses map onto stimuli (Fitts and Seeger, 1953). For example, rapidly pressing a given button in response to the flash of a given light is easier if corresponding lights and buttons have the same spatial arrangement. Compatibility effects lend themselves to ecological explanation as they arise from the ease with which a given stimulus-response relationship generalizes from preexisting dispositions, invoking the advantage of rapid and highly practiced or innate responses when they are suited to the context.

In the present case, the outcome of choice led to an equivalent result in both keep and discard frames: participants received one of the preferred foods. The attentional response nevertheless prominently reflected compatibility between the stimulus and framing condition, with subjects attending the less preferred item more during the discard condition. Because choosing an appetitive stimulus naturally entails stimulus-directed orienting and approach, one may also consider a second order of compatibility: that between the elicited attentional response and motivational context. In this respect, the discard condition should create a conflict of compatibility between attention and motivational context, which is absent in the keep condition. Effects related to this mode of compatibility offer a plausible and parsimonious explanation for two prominent differences in the gaze response between the conditions: the greatly reduced duration of the phase I response in the discard condition and the delayed phase II response in the keep condition. Both of these effects might be explained by compatibility-related suppression of process 1 in the discard condition, resulting in a more rapid disinhibition of process 2. Thus process 2 is influenced by compatibility between stimulus and framing condition, while process 1 is modulated according to the compatibility between the process 2 response and motivational context.

A future question is whether the opposite pattern of responses might emerge for aversive stimuli. Such a prediction follows if orienting towards aversive stimuli in the context of avoidance depends similarly on process 1. We have shown that two types of compatibility effects described here, between attention and response and between attention and motivational context, might be separately manipulated through framing to distinguish their contributions.

These findings mesh with emerging neurobiological accounts of attention and decision-making, which have converged on overlapping brain systems involving most notably the amygdala. The amygdala has a well established role in rapid orientation to biologically salient, especially threat-related stimuli (Davis, 1992; LeDoux, 2000), and associated emotional responses (Adolphs et al., 1995). Moreover, neural responses in amygdala represent stimulus value for both appetitive and aversive stimuli (Schoenbaum et al., 1998; Gottfried et al., 2003; Paton et al., 2006; Morrison and Salzman, 2010; Jenison et al., 2011) as well as state value (Belova et al., 2008), placing it at the nexus of systems that modulate attention and represent value, and positioning it to mediate effects of framing with respect to both attention and choice outcome (De Martino et al., 2006). Such a modulatory role is further supported by recent evidence from lesion work in macaques: while lesioning amygdala does not abolish the representation of value in prefrontal cortex, it results in delayed and diminished responses selectively in orbitofrontal cortex (OFC; Rudebeck et al., 2013). OFC and associated regions of ventromedial prefrontal cortex have been implicated in the encoding of relative stimulus value within a variation of the current task (Lim et al., 2011). Multiple strands of evidence therefore lead us to expect a role for amygdala in mediating attentional responses associated, in particular, with process 1, raising a number of questions about the nature of any contribution. In line with its role in CS–US conditioning, amygdala might encode associations that drive system 1 responses. Alternatively, it might modulate responses elsewhere according to motivational context, in line with its close association with other regions of medial temporal lobe. As a heterogeneous structure, it may also participant in multiple aspects of these functions.

Along with amygdala, other regions previously associated with emotion, such as medial prefrontal cortex and insula have also been implicated in framing (Knutson et al., 2008), highlighting the importance of emotional responses in framing and other biases of decision making. Emotions themselves, however, represent a complex interaction of behavioral dispositions and physiological responses, which can likely be further decomposed into more elementary mechanisms, including orienting, arousal, avoidance and approach behavior (Lang, 1995) as well as the representation of associated body states in sensory cortices (Damasio, 1994). A better understanding of the ecological and neurobiological origins of framing effects will require a fine-grained separation of the multiple processes that underlie emotions and their relationship to multiple processes that contribute to decision-making and attention (Seymour and Dolan, 2008).

A number limitations with the current study should be noted. First, we have proposed a model of non-overlapping, mutually competitive processes to explain our observations. An alternative possibility is suggested by the distinct effects of block and preference in the GLM (**Figure 4**): two concurrent processes that exert an additive influence on attention. Under this model the first process, corresponding to the effect of preference, must drive attention initially to the more preferred item and later to the less preferred item while the second task-dependent process, corresponding to the effect of framing condition, becomes steadily more engaged over time. This alternative model attributes greater complexity to the first process, whose effect in biasing attention must reverse from the more preferred stimulus to the less preferred over time, and also requires that the effects of the two processes happen to cancel initially in the discard condition and later in the keep condition so that no bias of gaze appears during respective periods. We therefore view this alternative model as considerably less parsimonious or probable than the first.

We also intentionally limited participants’ choices to foods they rated positively in order to restrict choices to appetitive options. Further work should consider whether similarly phasic mechanisms operate in choosing between aversive options. Finally, the current work was limited to repeated binary choices between two items, which may not reflect processes engaged in more realistic situations, perhaps better exemplified by a single choice between dozens of snacks in a vending machine or options in the store. Future research should expand beyond binary choice situations to examine whether the current evidence holds when considering multiple options simultaneously (Krajbich and Rangel, 2011).

## Conflict of Interest Statement

The authors declare that the research was conducted in the absence of any commercial or financial relationships that could be construed as a potential conflict of interest.
